# Transcriptome Changes of Hematopoietic Stem and Progenitor Cells in the Peripheral Blood of COVID-19 Patients by scRNA-seq

**DOI:** 10.3390/ijms241310878

**Published:** 2023-06-29

**Authors:** Jinfu Zhang, Xin Shu, Rong Deng, Zihao Yang, Manling Shu, Xiangying Ou, Xuan Zhang, Zhenyu Wu, Huihong Zeng, Lijian Shao

**Affiliations:** 1 Department of Occupational Health and Toxicology, School of Public Health, Nanchang University, Nanchang 330006, China; zjf99934@163.com (J.Z.); shuxin6786@163.com (X.S.); amstrong777@163.com (R.D.); yzh19962021@163.com (Z.Y.); 18373784338@163.com (M.S.); oxy508@163.com (X.O.); zx1775524@163.com (X.Z.); wzy9568@126.com (Z.W.); 2 Department of Histology and Embryology, School of Basic Medicine, Nanchang University, Nanchang 330006, China; zenghuihong@ncu.edu.cn

**Keywords:** COVID-19, single-cell RNA sequencing (scRNA-seq), hematopoietic stem and progenitor cells (HSPCs), immune response, COVID-19 vaccination

## Abstract

Coronavirus disease 2019 (COVID-19) threatens public health all over the world. It is well-accepted that the immune cells in peripheral blood are widely involved in the pathological process of COVID-19. However, hematopoietic stem and progenitor cells (HSPCs), as the main source of peripheral immune cells, have not been well studied during COVID-19 infection. We comprehensively revealed the transcriptome changes of peripheral blood HSPCs after COVID-19 infection and vaccination by single-cell RNA-seq. Compared with healthy individuals, the proportion of HSPCs in COVID-19 patients significantly increased. The increase in the proportion of HSPCs might be partly attributed to the enhancement of the HSPCs proliferation upon COVID-19 infection. However, the stemness damage of HSPCs is reflected by the decrease of differentiation signal, which can be used as a potential specific indicator of the severity and duration of COVID-19 infection. Type I interferon (IFN-I) and translation signals in HSPCs were mostly activated and inhibited after COVID-19 infection, respectively. In addition, the response of COVID-19 vaccination to the body is mild, while the secondary vaccination strengthens the immune response of primary vaccination. In conclusion, our study provides new insights into understanding the immune mechanism of COVID-19 infection.

## 1. Introduction

Since the outbreak of coronavirus disease 2019 (COVID-19) caused by severe acute respiratory syndrome 2 (SARS-CoV-2), COVID-19 has rapidly spread around the world, bringing unprecedented challenges to world public health. As of 18 June 2023, the number of confirmed cases of COVID-19 has exceeded 767 million, including more than 6.9 million death cases worldwide (WHO, https://www.who.int/data, accessed on 18 June 2023). Although the vaccination rate for COVID-19 is very high, there are still a considerable number of COVID-19 patients. Moreover, the recent emergence of the SARS-CoV-2 variant has enhanced the transmissibility and infectivity of COVID-19, which may lead to a more serious pandemic [1,2,3,4]. There is an urgent need to deeply understand the pathological mechanism of COVID-19 to better prevent and control this devastating pandemic.

The severity of COVID-19 patients varies from asymptomatic to death [5,6,7,8,9,10]. The virus usually infects the lungs causing fever and cough. In serious cases, it causes acute respiratory distress syndrome (ARDS) and respiratory failure, eventually leading to death [7,9,10]. The “cytokine storm” caused by excessive inflammation is one of the important causes of death upon COVID-19 infection [8,11,12]. The main clinical manifestations of COVID-19 patients are the increase of neutrophils and monocytes in the blood, the decrease of lymphocytes, and the increase of inflammatory cytokines, such as interleukin (IL)-6, IL-8, IL-10 and tumor necrosis factor α (TNF-α) [7,8,13,14]. Host immune response plays an important role in resisting COVID-19 infection. Many studies of transcriptomes in peripheral blood mononuclear cells (PBMCs) from COVID-19 patients have revealed that a variety of immune cells, such as T cells, B cells, natural killer cells, neutrophils, monocytes, macrophages and dendritic cells, were widely involved in the pathogenesis of COVID-19 patients [15,16,17,18,19,20,21,22]. Huang et al. found that dendritic cells, CD14^+^ monocytes, and megakaryocytes progenitor cells/platelets were expanded in patients with COVID-19 infection, along with a significant decrease of CD8^+^ T lymphocytes and natural killer cells in patients with critical condition [20]. More and more studies have also emphasized the importance of type I interferon (IFN-I) in response to COVID-19 infection [19,20,23]. The concentration of IFN-α in COVID-19-infected serum was significantly increased but decreased in critical patients [20,23]. Consistently, the expression level of IFN-I genes was elevated in patients with COVID-19 and negatively related to the severity of disease [24]. Moreover, translational- and ribosomal-related genes tend to decrease in various cell types with elevated IFN-I [24]. Therefore, previous studies extensively investigated the changes in mature immune cells (such as neutrophils and lymphocytes) in peripheral blood after COVID-19 infection. Hematopoietic stem and progenitor cells (HSPCs) can generate all these mature immune cells in peripheral blood. However, the changes in HSPCs during the COVID-19 infection were rarely studied, which prompted us to investigate the changes in HSPCs after the COVID-19 infection.

HSPCs are a group of cells with self-renewal and differentiation potential, which are essential to maintain the immune system and hematopoiesis hemostasis throughout the lifespan. When the body is challenged by a virus infection, it will promote HSPCs to enter the cell cycle, generating a large number of immune cells through proliferation and differentiation, which will meet the needs of immune responses [25]. In addition to exerting its hematopoietic function as precursors of immune cells, peripheral blood HSPCs can also produce cytokines and chemokines, such as TNF-α, TGF-β, IL6 and IL8 [26], and directly participate in host defense through their surface and intracellular receptors [27]. On the other hand, HSPCs express cell receptor angiotensin-converting enzyme 2 (ACE2) and transmembrane serine proteinase 2 (TMPRSS2), with which SARS-CoV-2 may enter and infect HSPCs [28]. Hector et al. reported that SARS-CoV-2 directly infects erythroid progenitor cells, leading to aberrant erythropoiesis [29]. Besides, the “cytokine storm” can damage to HSPCs [30]. These data indicate that HSPCs are involved in virus reaction upon COVID-19 infection, which might result in functional impairment of HSPCs. Even though the complex network of immune responses during COVID-19 infection has been widely studied, there is still a lack of comprehensive investigation on HSPCs after infection.

The present study mainly focused on CD34^+^ cells in peripheral blood, namely HSPCs, which are a small group of cells but critical for the immune system in our body. We, therefore, analyzed the transcriptome dynamics of HSPCs after SARS-CoV-2 infection and COVID-19 vaccination at the single-cell level. The present study of HSPCs is helpful in understanding the immune regulation process of peripheral blood in patients with COVID-19 infection in a more comprehensive way.

## 2. Results

### 2.1. HSPCs Acquisition and Sub-Clustering

**Figure 1:**
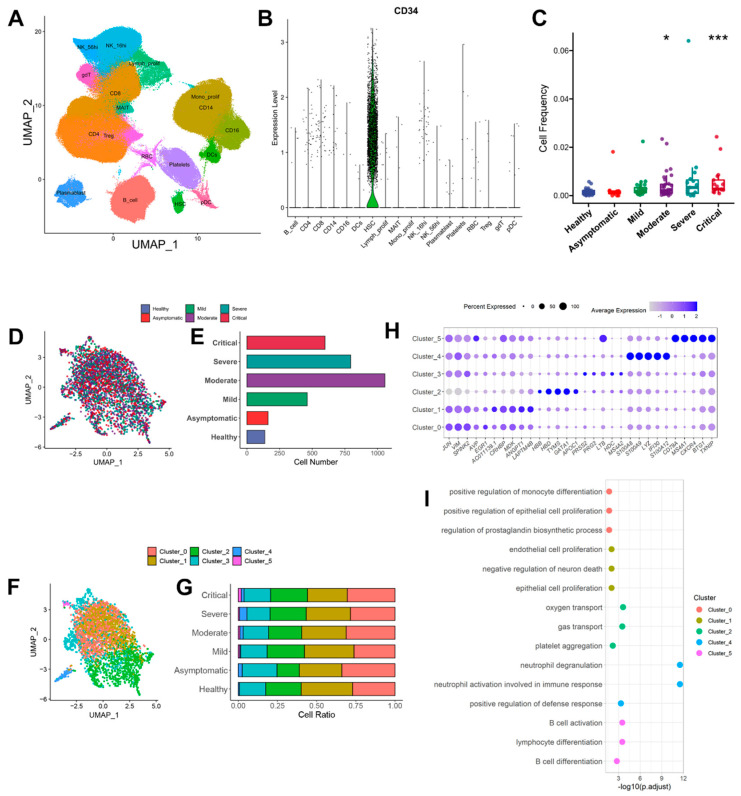
HSPCs acquisition and sub−clustering. (**A**) UMAP plot of all cell types in PBMCs. (**B**) Violin plot of CD34 expression in cell clusters. (**C**) Boxplot of HSPCs proportion in a healthy individual (*n* = 12) and COVID-19 patients with asymptomatic (*n* = 12), mild disease (*n* = 26), moderate disease (*n* = 32), severe disease (*n* = 15) and critical disease (*n* = 17). (**D**) Distribution of disease severity in HSPCs. (**E**) The total number of HSPCs in each group was used for the present study. (**F**) UMAP plot of the six cell clusters in HSPCs. (**G**) Percentage of Cluster 0~5 among different groups. The numbers of cells in Cluster 0~5 are 950, 911, 711, 526, 80 and 31, respectively. (**H**) Dotplot of the first five markers in Cluster 0~5. (**I**): GO-BP enrichment analysis of the top 10 markers of Cluster 0~5 and display the first three enriched terms. Cluster 3 has no enriched pathways. * *p* < 0.05, *** *p* < 0.001.

647366 PBMCs in Emily Stephenson’s study from 143 patients. Total cells were clustered into 18 populations based on gene expression patterns (Figure 1A). 3209 HSPCs (CD34^+^ cells) were extracted in the present study (Figure 1B). As shown in Figure 1C, the proportion of HSPCs in COVID-19 patients was elevated when compared to that in healthy individuals. The increments were more pronounced in those severe patients. Extracted HSPCs did not aggregate with severity, indicating the batch effect was well removed (Figure 1D). The total numbers of HSPCs in healthy, asymptomatic, mild, moderate, severe and critical cases were 137, 162, 462, 1056, 794 and 598, respectively, as shown in Figure 1E. HSPCs were further re-clustered, and 6 clusters (Cluster 0~5) were obtained (Figure 1F). As shown in Figure 1G, cells with similar biological significance, obtained by cell clustering based on the gene expression patterns, represented the proportion of each cluster in the different groups. Especially a new population appeared in COVID-19 groups (Cluster 5, Figure 1G), which highly expresses B cell-related genes, such as *CD79A* and *MS4A1* (Figure 1H). Subsequent biological process (BP) of gene ontology (GO) enrichment analysis showed that Cluster 5 was related to the activation and differentiation of B cells (Figure 1I). This new population might be associated with COVID-19 infection with the reduction of B cell numbers. The significance of Cluster 5 in COVID-19 patients warrants further investigation in the future.

### 2.2. Proliferation Signal of HSPCs Was Elevated in COVID-19 Patients 

**Figure 2:**
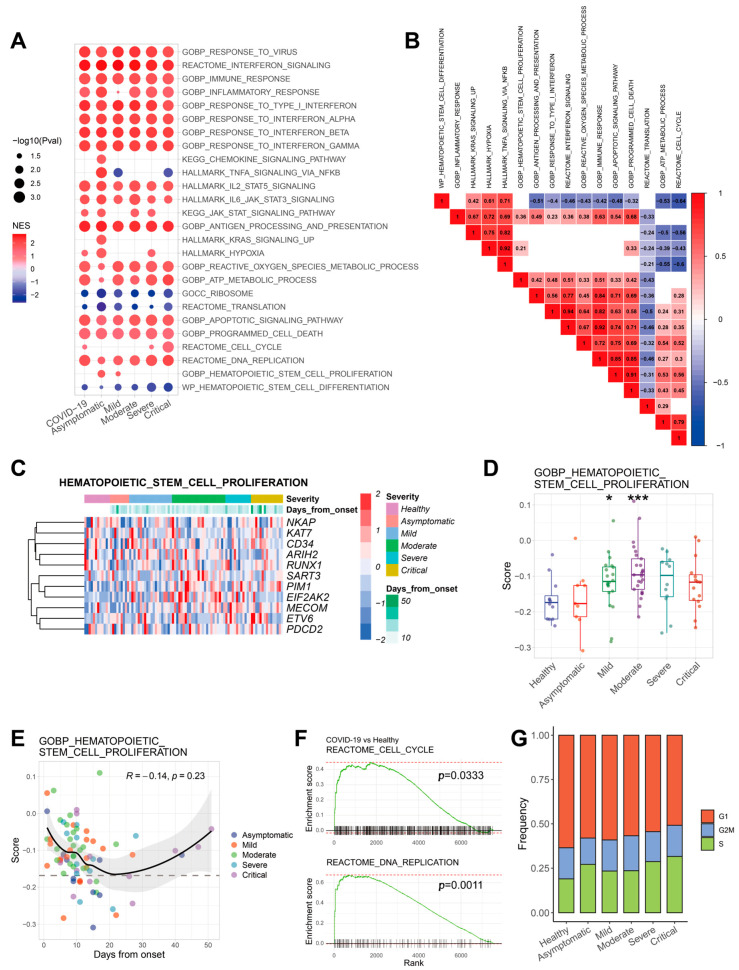
The proliferation signal of HSPCs was elevated in COVID-19 patients. (**A**) Dotplot of GSEA enrichment results. COVID-19 refers to all COVID-19 samples. The dots are all significantly enriched terms (*p* < 0.05). The size of the dot represents −log10 (pval). The red dots indicate that the term is activated in the COVID-19 group, while the green ones indicate that it is inhibited. The color depth indicates the normalized enrichment score (NES), which is used to evaluate the enrichment degree of the corresponding term. (**B**) Heatmap of correlations between 16 representative pathways. The correlation coefficient R was calculated by the Pearson correlation method. The pathways with *p* < 0.05 were displayed. (**C**) Heatmap of HSPCs proliferation-related genes expression. (**D**) Boxplot of HSC proliferation scores in each group. (**E**) Scatter plot of HSC proliferation score changes with days from infection onset in patients with COVID-19. The dashed black line marks the score of a healthy population. (**F**) GSEA plot of cell cycle and DNA replication. (**G**) Percentage bar plot of cell cycle phase proportion of each group. * *p* < 0.05, *** *p* < 0.001.

The transcriptome changes of HSPCs after COVID-19 infection were further investigated. To perform GSEA analysis, differential gene analysis was conducted between COVID-19 groups (asymptomatic, mild, moderate, severe and critical) and healthy groups, as shown in Figure 2A. GSEA analysis was performed according to the order of log2FC of each gene. The data from Figure 2A showed that the genes with multiple biological processes were up-regulated, such as immune response, inflammatory response, interferons, TNF-α signaling, antigen processing and presentation, cellular apoptosis and death, reactive oxygen species (ROS) metabolic process, hyperoxia, ATP metabolic process, cell cycle, DNA replication and hematopoietic stem cells (HSC) proliferation. However, those genes with differentiation and translation pathway in HSPCs were significantly downregulated after infection. As shown in Figure 2A, the immune-related responses, such as responses to virus and interference signal, were significantly activated in all severity groups, while the cell cycle signal was activated in the COVID-19 group but not in asymptomatic, mild and moderate groups. However, few signal pathways have a perfect linear relationship with severity. This might be because the regulatory network of HSPCs after COVID-19 infection is complex. These pathways could be affected by many endogenous and exogenous factors.

To quantify these signal pathways in single cells, pathway scores were calculated based on the expression of genes involved in corresponding pathways, along with conducting correlation analysis (Figure 2B). For example, the translation signal was in column 14, row 14; we can see that the pathways in rows 2 to 13 were negatively correlated with the translation signal, while row 15 was positively correlated with it. Specifically, the correlation coefficient between the translation signal (column 14) and the IFN-I signal (row 8) was −0.5. The correlation coefficient between the translation signal and ATP metabolic process signal (row 15) was 0.29.

Because the frequencies of HSPCs were increased post-infection, the changes in the proliferation-related pathways of HSPCs were first analyzed in peripheral blood. As shown in Figure 2C, the expression of HSC proliferation genes, such as PIM1, was increased in the COVID-19 group when compared to the healthy population (Figure S1A). Specifically, the HSC proliferation scores were initially increased and then slightly decreased with disease severity (Figure 2D). However, the correlation between HSC proliferation scores and the cell proportion was not significant (Figure S1B). These data suggest that there may be other ways to enhance the proliferative capacity and promote the generation of HSPCs.

In the present study, the days from infection onset were not related to the severity (not a confounding factor); the relationship between the infection time of COVID-19 and related signal pathways was directly observed (Figure S1C). The results showed that the HSC proliferation signal first decreased and then increased with the duration of infection (Figure 2E). The average level of HSC proliferation in the COVID-19 group was higher than that in the healthy group. Related pathways, such as cell cycle and DNA replication, were up-regulated in the COVID-19 group (Figure 2A,F), which is correlated with the increase in the proportion of S-phase cells in HSPCs (Figure 2G). Meanwhile, cell cycle scores were increased in moderate and critical groups (Figure S1D).

### 2.3. Differentiation Signal of HSPCs Was Inhibited in COVID-19 Patients 

**Figure 3:**
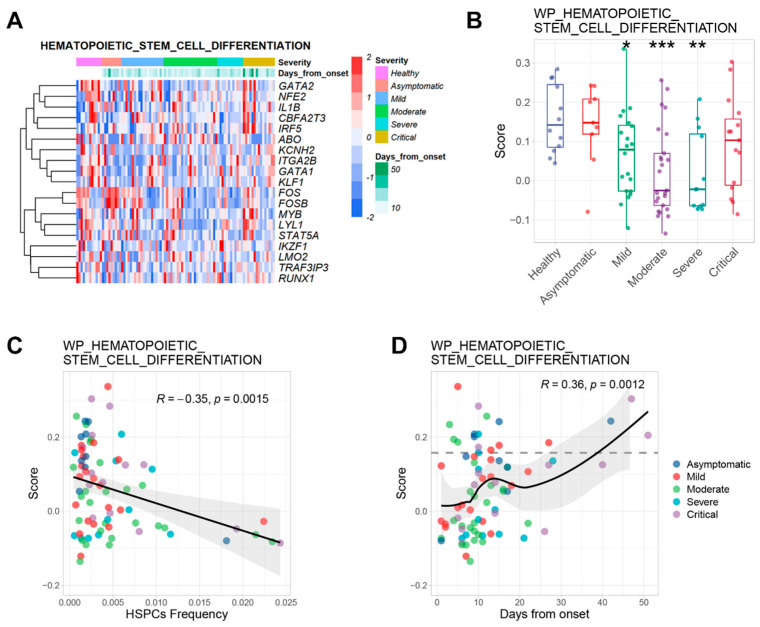
The differentiation signal of HSPCs was inhibited in COVID-19 patients. (**A**) Heatmap of HSC differentiation-related genes expression in each group. (**B**) Boxplot of HSC differentiation scores in each group. (**C**) Scatter plot of HSC differentiation score changes with HSPCs proportion. (**D**) Scatter plot of HSC differentiation score changes with days after onset in patients with COVID-19. * *p* < 0.05, ** *p* < 0.01, *** *p* < 0.001.

The changes in immune cells in peripheral blood depend on the proliferation and differentiation of HSPCs. GSEA data showed that the HSPCs differentiation signal was decreased in the COVID-19 group when compared to the healthy group (Figure 2A). HSPCs differentiation-related genes, such as GATA1 and GATA2, were significantly downregulated in the COVID-19 group (Figure S1E,F). In contrast to the proliferation signal, the differentiation signal was significantly decreased in milder patients and more stable in severe patients when compared to the healthy group (Figure 3B). Collectively, these data implied that the stemness of HSPCs might be compromised after COVID-19 infection. 

No correlation was found between proliferation and differentiation signals in HSPCs after infection (Figure 2B and Figure S1G). However, the differentiation signal was negatively correlated with the HSPCs proportion (Figure 3C), which indicates that the increase in the proportion of HSPCs was accompanied by the decline in their differentiation ability during COVID-19 infection. The differentiation signal might be related to the severity of COVID-19 infection due to the increment of HSPC proportion in COVID-19 patients.

The differentiation signal was negatively correlated with cellular apoptosis and ROS metabolism (Figure 2B), which may be the factors leading to the decrease of differentiation signal upon COVID-19 infection. The trend of differentiation score steadily increased with the days from infection onset. The differentiation signal was significantly inhibited at the early stage of infection and then rose with the days from the onset of COVID-19 (Figure 3D). Therefore, differentiation signal might play an important role during COVID-19 infection, which is not only related to the severity but also to the duration of infection. Overall, the HSPCs differentiation signal might be a specific indicator to reflect the severity and duration of COVID-19 infection.

### 2.4. IFN-I and Translational Signals Are the Most Significant Changes in HSPCs after COVID-19 Infection

**Figure 4:**
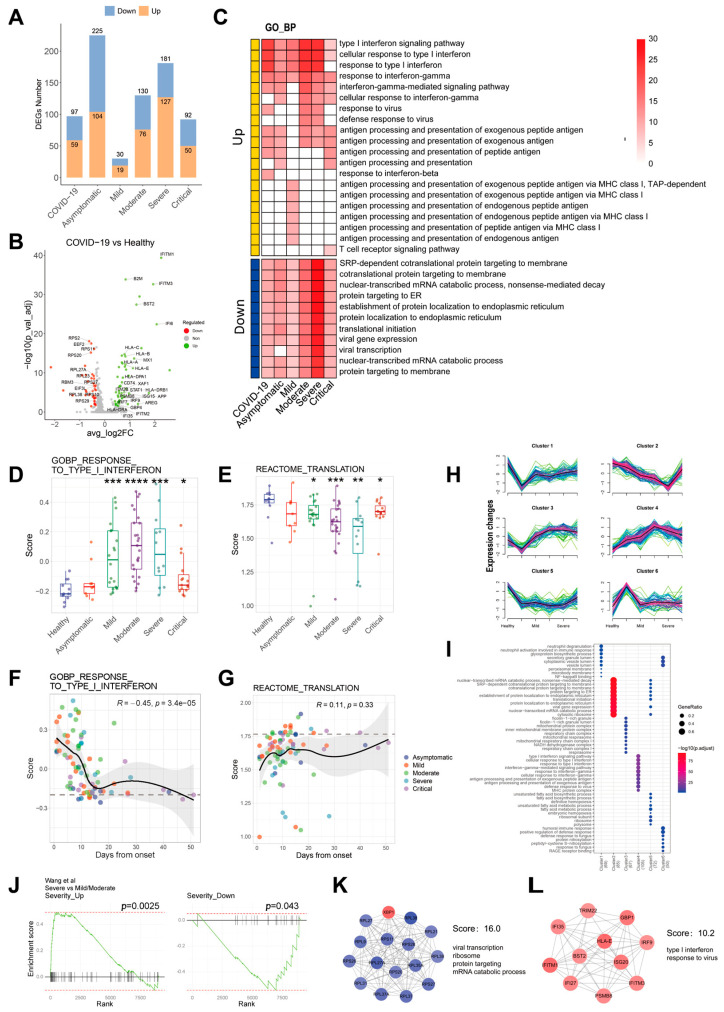
IFN-I and Translational signals are the most significant changes in HSPCs after COVID-19 infection. (**A**) Barplot of the numbers of DEGs in each group compared with the healthy group. The top and inside of the barplot are marked with the numbers of total DEGs and up-regulated genes, respectively. (**B**) Volcano plot of differential gene analysis between COVID-19 group and healthy group. Red dots represent down-regulated genes. Green dots represent up-regulated genes. The marked genes were those involved in pathways in (**C**). (**C**) Based on the enrichment of DEGs between cells from COVID-19 patients and cells from healthy individuals. The top ten up- and down-regulated GO-BP enrichment pathways were shown in each group. The column for COVID-19 represents the GO-BP results of DEGs when compared between COVID-19 patients and healthy individuals. (**D**,**E**) Boxplot of IFN-I (**D**) and translation (**E**) score in each group. (**F**,**G**) Scatter plots of IFN-I (**F**) and translation (**G**) score change days after onset in patients with COVID-19. (**H**) DEGs clustering according to the changing trend of disease severity. (**I**) First 10 GO-BPs of each cluster in (**H**). (**J**) Ninety severity-related genes can well distinguish the disease severity. (**K**,**L**) Two key gene modules of the PPI network of severity-related genes. Cluster 1 is related to ribosomes. Cluster 2 is related to IFN-I. Blue represents a negative correlation. Red represents a positive correlation. The color depth represents the absolute value of correlation coefficient R. * *p* < 0.05, ** *p* < 0.01, *** *p* < 0.001, **** *p* < 0.0001.

According to the differential gene analysis above, the differentially expressed genes (DEGs) were identified with *p*.adjust < 0.05 and |log2FC| > 0.35. Figure 4A quantitatively observes the changes in DEGs in COVID-19 patients with different severity when compared to a healthy population. The numbers of DEGs in the asymptomatic group were the highest, reaching 225, while that in the mild group was only 30 (Figure 4A). As shown in the volcano plot in Figure 4B, many genes related to virus infection or immunity, such as *IFI*s, *PSM*s and *HLA*s, were up-regulated in the COVID-19 group. However, translation or ribosome-related genes, such as *RPL*s, were down-regulated after COVID-19 infection (Figure 4B). GO-BP enrichment analysis, as shown in Figure 4C, was subsequently performed based on these DEGs. The top 10 up- and down-regulated terms for each group were shown in the heatmap. The up-regulated genes were mainly related to IFN-I signal, antigen processing and presentation. However, the down-regulated genes were related to nucleic acid transcription and protein translation, which was consistent with the results of GSEA (Figure 2A).

As a feature of COVID-19 infection, interferons had a decisive role in clinical severity. Our results showed that the IFN-I signal increased in the COVID-19 group and decreased in more severe groups compared to less severe groups (Figure 4D and Figure S2A). IFN-I signal was sharply decreased in the early stage, which may be related to the strong signal of IFN-I in the early stage (Figure 4E). Opposite to the IFN-I signal, the translational signal decreased in the COVID-19 group (Figure 4F and Figure S2B), which was not related to the duration post-infection. These data indicated that the translational level of HSPCs might not change over time but was mainly determined by the severity (Figure 4G). Further analysis indicated that the changes in IFN-I and translational signals were opposite, along with a significant negative correlation (Figure 2B).

To confirm the above result, the Mfuzz package was further used to determine the dynamic expression pattern of DEGs along with the severity of the disease. Six gene clusters were obtained (Figure 4H). The dynamic changes in cluster 2 and cluster 4 were consistent with those of IFN-I and translation signals upon COVID-19 infection, respectively. These data were further confirmed by GO-BP analysis (Figure 4I).

Additionally, Spearman rank correlation analysis was performed on all genes according to the order of Health < Asymptomatic < Mild < Moderate < Severe < Critical. A total of 90 genes with |R|> 0.25 and *p* < 0.05 were obtained, of which 69 were positively correlated with disease severity and 21 negatively correlated (Figure S2C). The top correlated genes are CLU (r = 0.453) and SOX4 (r = −0.375) (Figure S2D,E). The data provide a reference for revealing the transcriptome characteristics of HSPCs related to clinical manifestations. These severity-related genes were verified in the GSE165080 dataset, which proved their reliability (Figure 4J). The protein-protein interaction (PPI) network of these 90 severity-related genes was constructed by using the STRING online database and Cytoscape software (Figure S2F). The MCODE in Cytoscape was used for the PPI network. Identified two key gene clusters, which were related to IFN-I and translation signals (Figure 4K,L). Altogether, these results suggested that IFN-I and translation signals play a critical role in the dysfunctional HSPCs of COVID-19 patients, which are highly correlated with disease severity.

### 2.5. Effects of COVID-19 Vaccination on HSPCs

**Figure 5:**
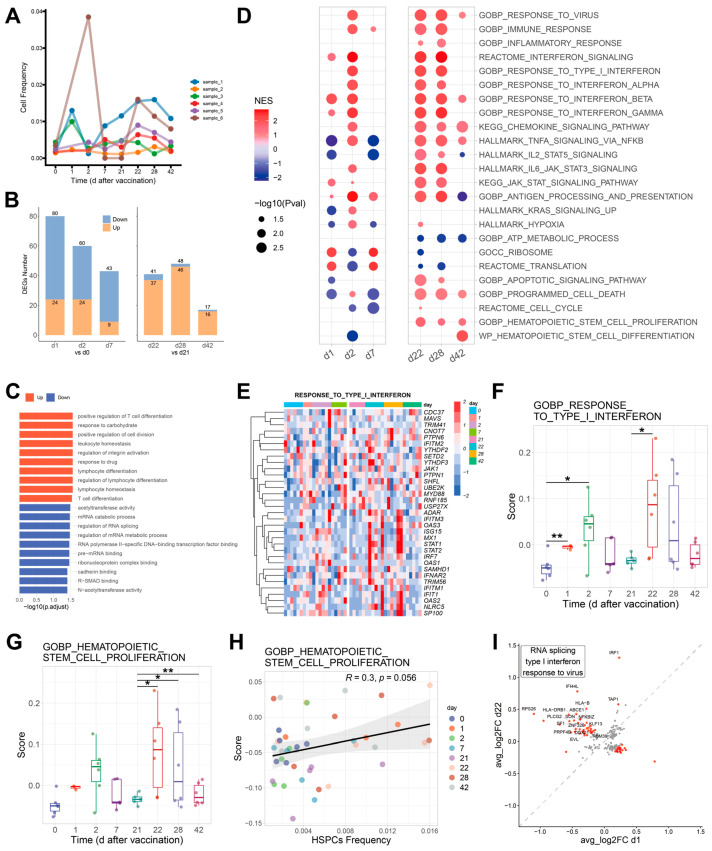
Effects of COVID-19 vaccination on HSPCs. (**A**) The curve of the HSPCs proportion of the six individuals changes with vaccination duration. The colors represent each individual. (**B**) Barplot of the number of DEGs after two vaccinations. (**C**) Barplot of GO enrichment pathways of DEGs after COVID-19 vaccination (d1 vs. d0). (**D**) Dotplot of GSEA after two vaccinatI. (**E**) Heatmap of IFN-I related genes expression. (**F**) Boxplot of IFN-I score in days after vaccination. (**G**) Boxplot of HSC proliferation score in days after vaccination. (**H**) Scatter plot between HSC proliferation score and HSPCs proportion. (**I**) Scatter plot of DEGs between d 22 and d1. The *p* value of all points in the Figure was less than 0.1. The red dot represents the genes whose log2FC difference between d 22 and d 1 is greater than 0.3. The up-regulated genes enrichment pathways in d 22 were labeled in the upper left corner. * *p* < 0.05, ** *p* < 0.01.

Vaccination is of great importance for the prevention of COVID-19 infection. Therefore, the GSE171964 dataset was chosen to observe changes in HSPCs with COVID-19 vaccination (Figure S3A,B). The proportion of HSPCs of 6 individuals on day 0 was similar, which proves the reliability of the data. On the first day after primary vaccination (day 1), the proportion of HSPCs significantly increased and then slowly decreased. On the first day after the second vaccination (day 22), the proportion of HSPCs began to increase again, proving that COVID-19 vaccination can immediately stimulate the expansion of peripheral blood HSPCs (Figure 5A).

Differential gene analysis showed that more DEGs appeared after primary vaccination than secondary vaccination, and more DEGs emerged on day one and day 22. Most of the DEGs were down-regulated after primary vaccination, which were up-regulated after secondary vaccination (Figure 5B). These data suggested that the effects of primary vaccination have not completely dissipated. The transcriptional responses of HSPCs were strengthened after secondary vaccination.

GO analysis conducted for DEGs between day one and day zero showed that the up-regulated genes were mainly related to lymphocyte or T cell differentiation, whereas the down-regulated genes were mainly related to RNA catabolic, metabolic, splicing and binding after vaccination (Figure 5C). The IFN-I signal was up-regulated on day 2 (two days after primary vaccination), day 22 (one day after secondary vaccination) and day 28 (seven days after secondary vaccination) (Figure S3C–G).

In order to observe the changes in pathways after vaccination, we selected the pathways from Figure 2A to conduct GSEA analysis on different days after two vaccinations. Several pathways were enriched after vaccination, indicating that the vaccination activated HSPCs. Furthermore, there were also differences in gene expression between primary vaccination and secondary vaccination. Apoptosis and programmed cell death signal were inhibited after primary vaccination and activated after secondary vaccination. ATP metabolism was down-regulated without changes in the translational signal. Notably, the HSPCs proliferation signal was significantly activated with minor changes in HSPCs differentiation in COVID-19 vaccination (Figure 5D).

The IFN-I signal was increased immediately after vaccination but decreased with time, which was enhanced after secondary vaccination (Figure 5E,F). The HSPCs proliferation signal significantly increased after secondary vaccination and did not significantly decrease days after vaccination (Figure 5G and Figure S3H). The HSPCs proliferation signal might promote the increase in the proportion of HSPCs (Figure 5H).

In order to further compare the changes between the two vaccinations, the common DEGs of d1 and d22 with *p* < 0.1 were included. The genes with log2FC differences between d1 and d22 greater than 0.3 were obtained (Figure 5H, red dot). Compared with d1, 41 genes were up-regulated in d22, and 20 genes down-regulated. These up-regulated genes were mainly related to RNA splicing, IFN-I signal and virus infection (Figure 5I), indicating that these signals were increased after secondary vaccination compared to primary vaccination.

Overall, the vaccination can increase the number of HSPCs, and enhance the immune response, such as activation of IFN-I signal and proliferation ability of HSPCs. The enhancement of immune response by secondary vaccination was stronger than that by primary vaccination.

## 3. Discussion

As a supplementary source of immune cells after being consumed, many common manifestations of peripheral blood in COVID-19 patients, such as lymphocyte reduction and monocyte elevation, may be directly related to HSPCs. In addition, HSPCs themselves are an important part of the immune response. It has been reported that HSPCs will participate in the response even if there is no hematopoietic demand [31]. Therefore, whether it is used as a predictor of immune status and severity of patients or how it plays a potential role and its mechanism after COVID-19 infection deserves further investigation. 

We comprehensively described the transcriptome changes of HSPCs after COVID-19 infection. Compared with healthy individuals, the proportion and proliferation signal of HSPCs were activated after COVID-19 infection, while the differentiation signal was significantly restricted. The immune responses, especially the IFN-I signal, were significantly activated in HSPCs with COVID-19 infection. However, the translational signal was inhibited after COVID-19 infection. These findings suggest that HSPCs are functionally impaired after COVID-19 infection but still actively participate in the protective immune response.

Our study shows that the frequencies of HSPCs were increased in COVID-19 patients. This is consistent with a recent study that revealed that the increase in the frequency of HSPCs was an obvious feature of COVID-19 [32]. In particular, a new Cluster 5 was found only in severe patients in the present study. Expression of B cell-related genes was significantly increased in Cluster 5, which is involved in the activation and differentiation of B cells. Although Cluster 5 is a rare population in severe patients, the significance of Cluster 5 in COVID-19 patients warrants further investigation in the future. We also found the activation of proliferation and cell cycle after COVID-19 infection. An ex vivo study showed that the expansions of HSCs, multipotent progenitors (MPPs), common myeloid progenitors (CMPs)/megakaryocyte erythroid progenitors (MEPs) and granulocyte-macrophage progenitors (GMPs) were reduced after exposure to the S protein of SARS-CoV-2 [33]. It would be a plus to show that HSPCs in COVID-19 patients have higher proliferation ability than that in healthy populations through proliferation assay. However, it is difficult to measure HSCPs proliferation because of the rare numbers of HSPCs in peripheral blood. It is also challenging to obtain clinical peripheral blood samples with COVID-19 infection at this stage. This point will be tested through proliferation assay in the future. 

Our data and those of other research groups suggest that the expansion of HSPCs in peripheral blood is related to many factors, such as direct virus infection, hematopoietic balance and immune regulation. The extent to which HSPCs can be infected by the COVID-19 virus and the complex relationship between HSPCs infection and peripheral blood immunity deserve further investigation. Our data indicate that differentiation of HSPCs may be impaired after COVID-19 infection. Surprisingly, the differentiation signature is closely related to the severity and duration of COVID-19 infection. These results suggest that HSPCs are sensitive to the infection, which may be an effective predictor of the prognosis of COVID-19 patients. Aaron J. Wilk et al. found that HSPCs in severe and fatal COVID-19 cases had myeloid skewing differentiation feature [34]. The differentiation lineage of HSPCs needs to be further observed and confirmed according to the changes in the proportion of Its progenitor cell subsets (such as HSCs, MPPs and hematopoietic progenitor cells). The relationship between HSPCs differentiation and peripheral blood mature immune cells deserve further study. However, due to the limited number of cells in this study, the subclusters were not well divided into classical populations, but we did find a small cluster (Cluster 5) in more serious cases, which may be related to the decrease of lymphocytes upon COVID-19 infection.

Among many inflammatory mediators produced by COVID-19 infection, interferons have received extensive attention. Interferons are essential for antiviral immunity and are increased in the serum of patients with COVID-19 [35]. Interferon signal in various types of cells in peripheral blood is generally increased, including IFN-I (IFN-α and IFN-β) and IFN-II (IFN-γ). Consistent with the previous results, we found that the IFN-I signal increased in the COVID-19 group but was negatively related to the severity of COVID-19. These data suggest that IFN-I is a common response in peripheral blood cells after COVID-19 infection. IFN-I signaling can regulate the activation of HSPCs, promote HSPCs to enter the cell cycle, and induce cell apoptosis and death [36,37]. Previous studies have shown that exposure to IFN-I can induce rapid and transient proliferation of HSPCs [36]. Consistently, both HSPCs proliferation and IFN-I signals were at high levels in the early stage of infection. In the present results, the IFN-I signal had the highest correlation with translation. Previous studies showed that polyinosinic-polycytidylic acid treatment, an inducer of IFN-I signaling, significantly increased HSPCs proliferation in mice through activating intracellular STAT1 signaling, which negatively affected HSPCs engraftment ability [38,39]. Granulocyte colony-stimulating factor (G-CSF) treatment on mice stimulates HSPCs proliferation with loss of self-renewal ability and exhaustion of HSPCs [40]. The present study demonstrated that COVID-19 infection increases the proportion of HSPCs in peripheral blood by promoting HSPCs proliferation. The increment of proliferation after COVID-19 infection might lead to HSPCs functional defects, such as myeloid-biased differentiation. Our data demonstrated that COVID-19 infection blocked the translational signal in HPSCs. A previous study has shown that HSPCs had the property with high translational efficiencies of mRNA to maintain functional HSPCs [41]. Abnormal activation of the mTOR signal leads to increased myeloid lineage cells through aberrant translational reprogramming in HSPCs [41]. Another study demonstrated that proper ribosome assembly was important in the HSPCs maintenance and regeneration [42]. Therefore, blocking translational signals upon COVID-19 infection might contribute to the functional defect in HSPCs. 

COVID-19 vaccination can effectively prevent COVID-19 infection by stimulating immune cells. Consistent with COVID-19 infection, the proportion of HSPCs increased after vaccination, which may be driven by HSC proliferation. However, the differentiation signal was not significantly inhibited after COVID-19 vaccination. Immune responses, such as interferon and inflammation, were activated after vaccination. The responses to the secondary vaccination are more obvious than that to the primary vaccination. Our results proved the effectiveness of the vaccine in simulating COVID-19 infection and the necessity of booster vaccination.

Therefore, we described the clinical-related dynamic changes of HSPCs after COVID-19 infection at the single-cell level. These data further enriched the understanding of the physiological mechanism of peripheral blood after COVID-19 infection and vaccination. The inadequacy of the present study is that it failed to compare COVID-19 infection with other viral infections. Therefore, it is not clear whether these changes are general changes after being stimulated by viruses or specific to COVID-19 infection. 

## 4. Materials and Methods

### 4.1. HSPCs Single Cell RNA Sequencing (scRNA-seq) Data Acquisition

To determine the impact of COVID-19 infection on HSPCs, we presently extracted HSPCs by using a traditional marker CD34 in PBMCs from Emily Stephenson’s scRNA-seq data [18]. This HSPCs dataset includes 3297 cells from 143 samples, which have 24 healthy individuals, 5 individuals with non-COVID-19 severe respiratory illness (Non_covid19) and 12 healthy volunteers who were intravenously injected with lipopolysaccharide (LPS_10hours/90mins) and COVID-19 patients with asymptomatic (*n* = 12), mild diseases (*n* = 26), moderate diseases (*n* = 32), severe diseases (*n* = 15) and critical diseases (*n* = 17). Only 88 cells were defined from LPS_10hours/90mins and Non_covid19 groups, which were discarded in this study. Therefore, a total of 3209 HSPCs were used for the analyses. Another scRNA-seq data GSE165080 from Gene Expression Omnibus (GEO) database was also used in this study. The GSE165080 dataset contains 282 HSPCs from 11 healthy samples, 5 asymptomatic individuals, 13 moderate patients, 10 severe patients, 10 patients recovered from severe diseases and 3 samples collected at two different time points during hospitalization [43].

To analyze the effects of vaccination on HSPCs, 1071 HSPCs were obtained from dataset GSE171964, which includes 45 PBMC samples from 6 individuals [44]. To enrich dendritic cells, dendritic cells and total PBMCs were mixed in a ratio of 1:2 in this study. Each individual was inoculated with the BioNTech BNT162b2 mRNA vaccine on days 0 and 21. PBMCs of each individual on days 0, 1, 2, 7, 21, 22, 28 and 42 were collected for scRNA-seq.

### 4.2. Unsupervised Clustering and Cluster Marker Analyses

The scRNA-seq data was processed following Seurat (4.0.2) workflow. According to the original analysis, the first 20 integrated principal components (“pca_harmony”) were used to construct a weighted shared nearest neighbor (SNN) network. Clusters were found based on the graph-based clustering method with the resolution set to 0.5. The clustering results were analyzed and visualized through uniform manifold approval and project (UMAP). The marker genes in each cluster were identified by “FindAllMarkers” with the default parameters. Biological process (BP) of gene ontology (GO) analysis was conducted by clusterProfiler package (3.18.1) according to the first 10 up-regulated marker genes. The first three enriched terms were shown in dotplot using the ggplot2 package (3.3.2).

### 4.3. Analyses of Differentially Expressed Genes (DEGs) and Functional Enrichment

The analyses of differentially expressed genes (DEGs) between groups were performed using “FindMarkers”. The screening criteria for DEGs was |log2FoldChange (FC)|> 0.35, adjusted *p* < 0.05 and >10% of cells expressed in at least one group. To further understand the functions and pathways during COVID-19 infection, gene set enrichment analysis (GSEA) and GO analysis was performed based on gene expression differences. GSEA was performed by fgsea package (1.16.0) according to the rank of log2FC from high to low. H: hallmark genesets, C2: cultivated genesets and C5: GO genesets from the msigdbr package (7.5.1) were selected as reference genesets. Specific enrichment results with *p* < 0.05 are visualized by dotplot or GSEA plot. The clusterProfiler package (3.18.1) was used to perform GO analysis, which includes a biological process (BP), molecular function (MF) and cell composition (CC). The significantly enriched terms were identified by adjusted *p* < 0.05 and visualized by heatmap or dotplot.

### 4.4. Cell Scoring and Cell Cycle Phase Analyses

The leading edge genes obtained in the GSEA results of the different groups were used as reference genesets to score the corresponding terms of cells using the “AddModuleScore” of Seurat. The scores of specific terms in each sample were summarized by the average scores of cells. Samples with less than 3 cells were discarded, and 97 individuals were finally obtained. Similarly, the gene expression of each sample was calculated. Individual scores or gene expressions between groups were visualized with boxplot and compared by Student’s *t* test using “stat_compare_means” of the ggpubr package (0.4.0). The curves of scores with days from the onset were fitted by the “geom_smooth” of ggplot2, and the “stat_cor” of ggpubr was used for the Pearson correlation test. The average scores of healthy individuals were marked with black dashed. The correlations between scores of specific terms were analyzed with the Pearson correlation test and visualized by corrplot package (0.90). The pairs of terms with *p* < 0.05 were displayed. The cells can be divided into S, G2M and G1 cell cycle phases using “CellCycleScoring” according to the expression of cell cycle gene markers. The proportion of each phase in each group of cells was drawn by a percentage bar plot.

### 4.5. Dynamic Changes of DEGs with Disease Severity

Mfuzz package (2.50.0) was used to identify DEGs dynamic changes with disease severity. The gene expression of different disease severity was obtained by calculating the individual mean expression corresponding to the severity. The DEGs were divided into 6 different dynamic change patterns after preprocessing of missing or abnormal values and standardization according to Mfuzz instructions. GO analysis was conducted for the 6 patterns, and the top ten enriched terms were shown in the dotplot.

### 4.6. Correlations between Genes and Disease Severity

We used Spearman correlation analysis to further observe whether there were some genes associated with disease severity. The disease severity was sorted according to Health < Asymptomatic < Mild < Moderate < Severe < Critical. According to the individual gene expression, the correlations between gene expression and disease severity were calculated by the Spearman correlation method and genes with |R| > 0.25 and *p* <0.05 were identified as severity-related genes. STRING online database (https://string-db.org/, accessed on 18 June 2023) and Cytoscape software (3.8.1) were used to construct and visualize the protein-protein interaction (PPI)network between severity-related genes. The MCODE of Cytoscape was used to determine key gene modules.

### 4.7. Statistical Analysis 

All data processing and statistical analysis were conducted using R software (4.0.2). Shapiro Wilk test was utilized to determine whether the data in various groups conformed to normality. For the values that meet the normal distribution, *t* test was conducted to compare the differences between groups. Otherwise, the Wilcox test was used. Use Person or Spearman correlation tests for correlation analysis. All statistical *p*-values are bilateral, with Type I error α = 0.05.

## 5. Conclusions

The present data demonstrate that HSPCs played an important role during the process of COVID-19 infection, which enables us to further understand the potential mechanisms of severe inflammatory explosion. Multiple immune-related pathways in HSPCs were activated with the increased HSPCs proportion, while the differentiation ability of HSPCs might be damaged. Importantly, IFN-I inhibited the translational process and, thus, which may block virus replication in HSPCs after COVID-19 infection. Additionally, the COVID-19 vaccine can rapidly stimulate the expansion of HSPCs along with transcriptome changes in HSPCs after COVID-19 vaccination, which was much milder than that after COVID-19 infection. The detailed changes in HSPCs during COVID-19 infection still need further investigation.

## Data Availability

The datasets used and/or analyzed during the current study are available from the corresponding author upon reasonable request. In this study, publicly available datasets were analyzed. These data are available in Array Express under accession number E-MTAB-10026 (http://www.ebi.ac.uk/arrayexpress/experiments/E-MTAB-10026/, accessed on 18 June 2023) and NCBI GEO (GSE165080, GSE171964).

## Supplementary Materials

The following supporting information can be downloaded at: https://www.mdpi.com/article/10.3390/ijms241310878/s1.
