# Supplementary material for: Transcriptome Changes of Hematopoietic Stem and Progenitor Cells in the Peripheral Blood of COVID-19 Patients by scRNA-seq
Source: Int J Mol Sci. 2023 Jun 29;24(13):10878. doi: 10.3390/ijms241310878 (PMC10341542; doi:10.3390/ijms241310878)
Supplement: Supplementary file 1 [file ijms-24-10878-s001.zip › ijms-2430811-SI.pdf]

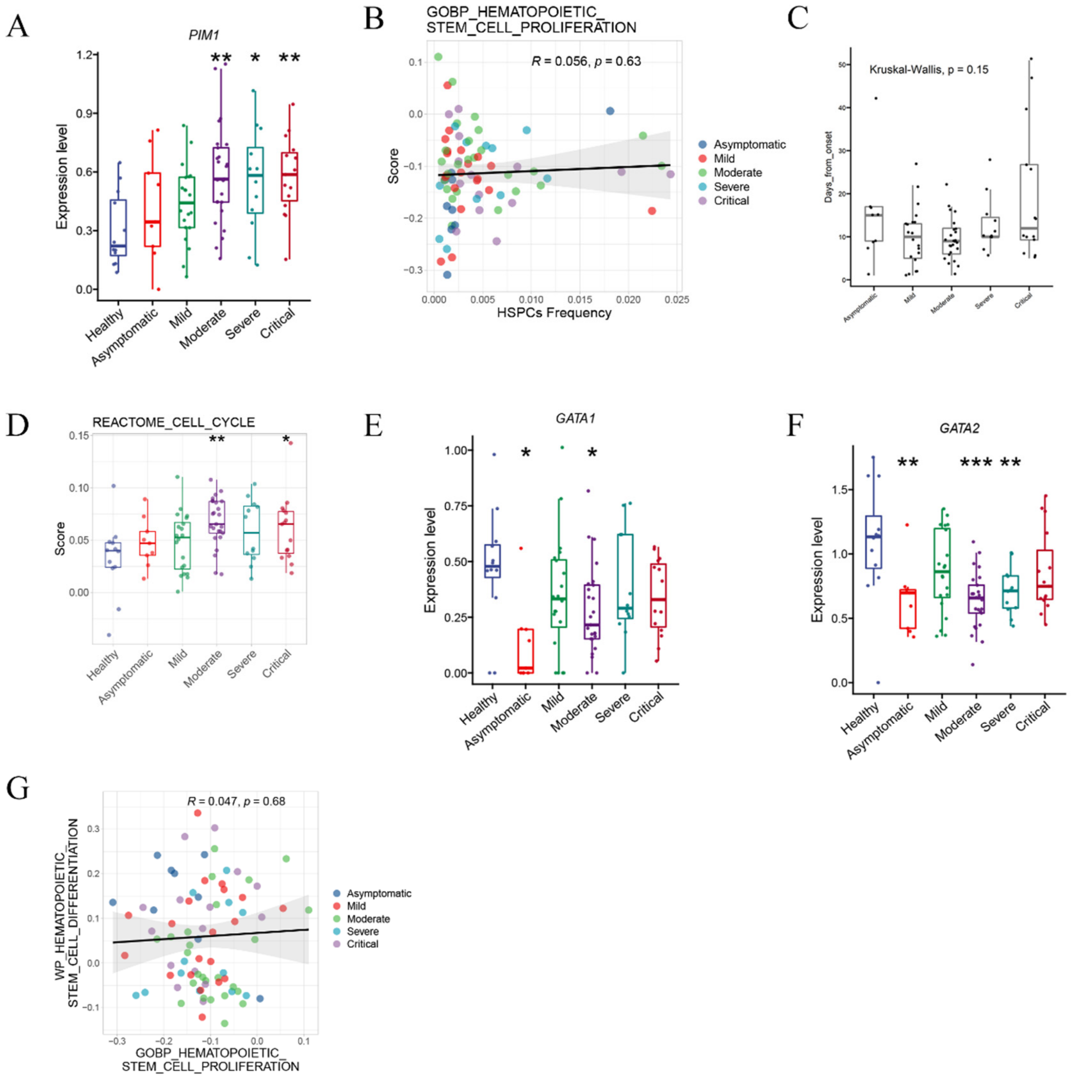

Figure S1. (A) Boxplot of PIM1 expression in each group. (B) Scatter plot of HSPCs proliferation score changes with HSPCs proportion. (C) Boxplot of days from the onset of each group. (D) Boxplot of cell cycle scores in each group. (E) Boxplot of GATA1 expression in each group. (F) Boxplot of GATA2 expression in each group. (G Scatter plot between HSPC proliferation and HSC differentiation scores.

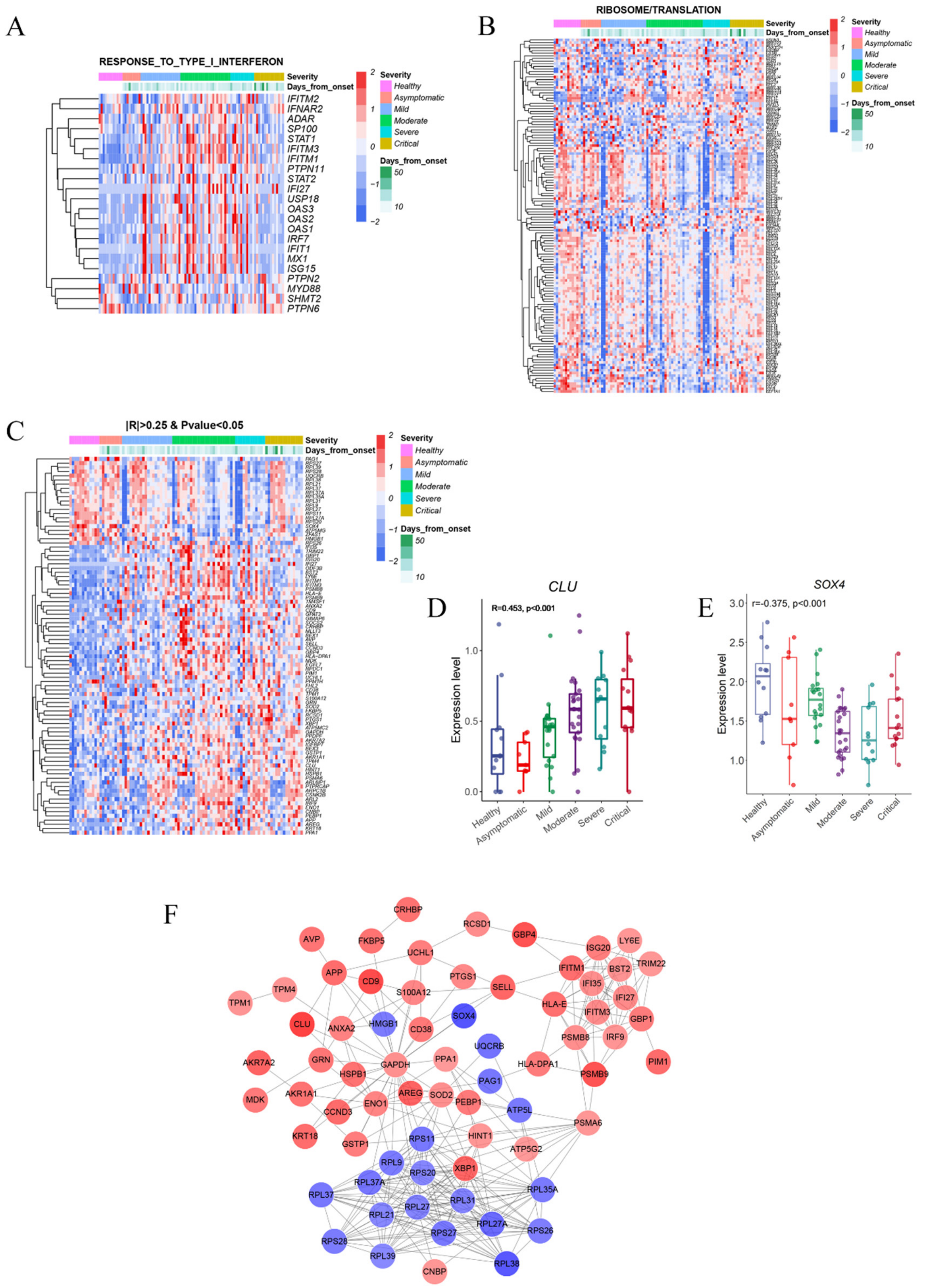

Figure S2. (A) Expression heatmap of leadingEdge genes in response to IFN-I pathway. (B) Expression heatmap of leadingEdge genes in ribosome/translation pathway. (C) Expression heatmap of severity related genes. (D) Boxplot of the expression of the most significant positive correlation gene CLU in each group. (E) Boxplot of the expression of the most significant negative correlation gene SOX4 in each group. (F) PPI network of severity-related genes. Blue represents negative correlation, red represents positive correlation. Color depth represents the absolute value of correlation coefficient.

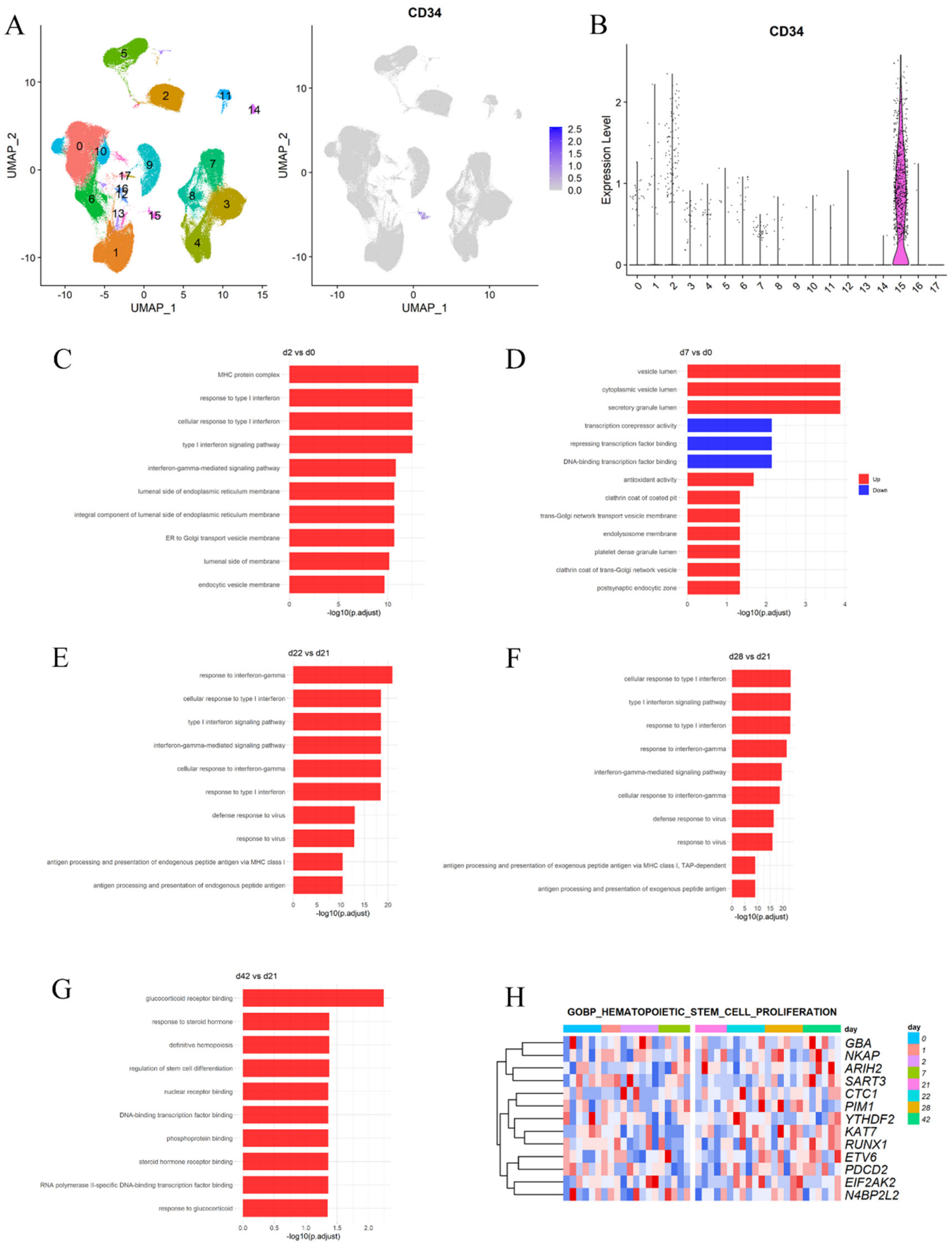

Figure S3. (A) UMAP plot of PBMCs in GSE171964. The total cells were divided into Cluster 0-17. Cluster 15 highly expresses CD34. (B) Violin plot of CD34 expression in Cluster 0-17. (C, D) Enrichment analysis of DEGs between day 2 (C) and day 7 (D), and day 0, respectively. (E-G) Enrichment analysis of DEGs between day 22 (E), day 28 (F), day 42 (G), and day 21, respectively.
